# Role of pulmonary microvascular endothelial cell apoptosis in murine sepsis-induced lung injury *in vivo*

**DOI:** 10.1186/s12931-015-0266-7

**Published:** 2015-09-16

**Authors:** Sean E. Gill, Marta Rohan, Sanjay Mehta

**Affiliations:** Centre for Critical Illness Research, Lawson Health Research Institute, London Health Sciences Center, London, ON Canada; Division of Respirology, Schulich School of Medicine and Dentistry, Western University, London, ON Canada; Department of Medicine, Schulich School of Medicine and Dentistry, Western University, London, ON Canada; Department of Physiology and Pharmacology, Schulich School of Medicine and Dentistry, Western University, London, ON Canada; Division of Respirology, E6.204, London Health Sciences Center – Victoria Hospital, 800 Commissioners Road East, London, ON N6A 5W9 Canada

## Abstract

**Background:**

Sepsis remains a common and serious condition with significant morbidity and mortality due to multiple organ dysfunction, especially acute lung injury (ALI) and acute respiratory distress syndrome (ARDS). Sepsis-induced ALI is characterized by injury and dysfunction of the pulmonary microvasculature and pulmonary microvascular endothelial cells (PMVEC), resulting in enhanced pulmonary microvascular sequestration and pulmonary infiltration of polymorphonuclear leukocytes (PMN) as well as disruption of the normal alveolo-capillary permeability barrier with leak of albumin-rich edema fluid into pulmonary interstitium and alveoli. The role of PMVEC death and specifically apoptosis in septic pulmonary microvascular dysfunction *in vivo* has not been established.

**Methods:**

In a murine cecal ligation/perforation (CLP) model of sepsis, we quantified and correlated time-dependent changes in pulmonary microvascular Evans blue (EB)-labeled albumin permeability with (1) PMVEC death (propidium iodide [PI]-staining) by both fluorescent intravital videomicroscopy (IVVM) and histology, and (2) PMVEC apoptosis using histologic fluorescent microscopic assessment of a panel of 3 markers: cell surface phosphatidylserine (detected by Annexin V binding), caspase activation (detected by FLIVO labeling), and DNA fragmentation (TUNEL labeling).

**Results:**

Compared to sham mice, CLP-sepsis resulted in pulmonary microvascular barrier dysfunction, quantified by increased EB-albumin leak, and PMVEC death (PI+ staining) as early as 2 h and more marked by 4 h after CLP. Septic PMVEC also exhibited increased presence of all 3 markers of apoptosis (Annexin V+, FLIVO+, TUNEL+) as early as 30 mins – 1 h after CLP-sepsis, which all similarly increased markedly until 4 h. The time-dependent changes in septic pulmonary microvascular albumin-permeability barrier dysfunction were highly correlated with PMVEC death (PI+; *r* = 0.976, *p* < 0.01) and PMVEC apoptosis (FLIVO+; r = 0.991, *p* < 0.01). Treatment with the pan-caspase inhibitor Q-VD prior to CLP reduced PMVEC death/apoptosis and attenuated septic pulmonary microvascular dysfunction, including both albumin-permeability barrier dysfunction and pulmonary microvascular PMN sequestration (*p* < 0.05). Septic PMVEC apoptosis and pulmonary microvascular dysfunction were also abrogated following CLP-sepsis in mice deficient in iNOS (*Nos2*^*−/−*^) or NADPH oxidase (*p47*^*phox−/−*^ or *gp91*^*phox−/−*^) and in wild-type mice treated with the NADPH oxidase inhibitor, apocynin.

**Conclusions:**

Septic murine pulmonary microvascular dysfunction *in vivo* is due to PMVEC death, which is mediated through caspase-dependent apoptosis and iNOS/NADPH-oxidase dependent signaling.

## Introduction

Sepsis remains a common and serious clinical problem with significant morbidity and mortality. It is estimated that one million cases of sepsis occur annually in North America, resulting in death in 20–30 % of cases, such that sepsis is the most common cause of mortality in Intensive Care Units (ICUs) and hospitalized patients [1–3]. Moreover, sepsis is a significant healthcare burden, as it consumes up to 45 % of total ICU costs [2, 4]. Morbidity/mortality in sepsis are principally due to injury and dysfunction of multiple organs, most commonly acute lung injury (ALI)/acute respiratory distress syndrome (ARDS) [2, 4–6]. Advances in our understanding of the pathophysiology of sepsis and organ dysfunction through basic and clinical research efforts have not significantly improved sepsis outcomes, as the treatment of sepsis and related organ dysfunction remains largely supportive care [6–8]. Indeed, clinical trials of novel anti-inflammatory therapeutic approaches have been disappointingly negative, as none of these therapies has improved clinical outcomes in sepsis and septic ARDS [6, 9–11].

The pathophysiology of ALI/ARDS in sepsis is the complex result of the actions of circulating cellular elements (e.g. polymorphonuclear leukocytes [PMN], platelets) and soluble inflammatory mediators, such as lipopolysaccharide (LPS) and multiple cytokines (e.g. tumour necrosis factor [TNF]α, interleukin [IL]1β), on multiple pulmonary cellular targets [7, 8, 12]. These include tissue-resident inflammatory cells (e.g. alveolar macrophages), alveolar epithelial cells, and the pulmonary vasculature, both large vessels and especially the pulmonary microvasculature. Septic microvascular dysfunction of both pulmonary and systemic vascular beds is clinically important as it is present early in the course of human sepsis and is associated with higher mortality [13–15], especially if persistent over time [16].

Pulmonary microvascular injury and resulting dysfunction are characterized by increased pulmonary microvascular PMN sequestration/adhesion as well as disruption of the normal pulmonary microvascular alveolo-capillary permeability barrier, resulting in extra-vascular leak of protein-rich edema and PMN into pulmonary interstitial and alveolar compartments and clinically severe hypoxaemic respiratory failure [8, 17–23]. Septic pulmonary microvascular dysfunction is primarily the result of the effects of septic inflammation on pulmonary microvascular endothelial cells (PMVEC) [7, 13, 23–29]. There are several potential mechanisms of septic PMVEC injury and dysfunction, including disruption of inter-cellular junctions (e.g. adherens junctions), microtubule activation, and actin cytoskeleton remodeling leading to cell retraction and gap formation [30–33]. Our previous work in a murine sepsis model identified that pulmonary microvascular/PMVEC barrier dysfunction, as reflected by enhanced albumin leak and oxidant stress required the presence of both alveolar macrophages and PMN, CD18-dependent PMN-PMVEC adhesion, increased nitric oxide (NO) production from inducible NO synthase (iNOS), and iNOS/NADPH oxidase-dependent peroxynitrite-mediated signaling [17–19, 34–36].

Recently, we reported the first direct *in vivo* evidence of septic PMVEC death, as observed by intravital videomicroscopy (IVVM) in septic mice [37]. Moreover, this septic PMVEC death was associated with evidence of apoptosis, as reflected by increased Annexin V binding, caspase activation, and TUNEL staining. PMVEC apoptosis has been proposed in sepsis models [38, 39], and could contribute to pulmonary microvascular albumin-permeability barrier dysfunction [40, 41]. Indeed, *in vivo* manipulation of various apoptosis pathways (e.g. the Fas-Fas ligand pathway) in animal models of sepsis improved measures of ALI severity suggesting a potential importance of global apoptosis in septic ALI [42, 43]. However, the specific role of PMVEC death in sepsis-induced ALI and septic pulmonary microvascular/PMVEC dysfunction remains uncertain, as does the importance of apoptosis in PMVEC death/dysfunction.

Thus, to further our previous studies, we first defined the time course of the onset of septic pulmonary microvascular barrier dysfunction *in vivo* in the murine cecal ligation/perforation (CLP) model of sepsis, and correlated this with the time course of PMVEC death as well as 3 specific molecular markers of PMVEC apoptosis. In addition, we assessed the effects of a specific, irreversible broad-spectrum caspase inhibitor, Q-VD, on septic PMVEC apoptotic death and septic pulmonary microvascular barrier dysfunction. Finally, we assessed the contribution of iNOS- and NADPH-oxidase-dependent oxidant stress to both septic PMVEC barrier dysfunction and death/apoptosis. Pulmonary microvascular Evans blue (EB)-labeled albumin leak increased by 2 h and peaked 4 h after CLP-induced sepsis, in parallel to and directly correlated with significant septic PMVEC death *in vivo*, as well as, and specifically, apoptosis. Moreover, effective inhibition of PMVEC apoptosis using the broad-spectrum caspase inhibitor, Q-VD, significantly attenuated septic pulmonary microvascular dysfunction, including both septic albumin hyper-permeability and pulmonary microvascular PMN sequestration. Both septic PMVEC apoptosis and pulmonary microvascular/PMVEC dysfunction were mediated through iNOS- and NADPH oxidase-dependent signaling.

## Materials and methods

### Ethics statement

All experimental animal protocols were performed in accordance with the Canadian Council on Animal Care guidelines for the care and handling of animals. The institutional Animal Care and Use Committee approved all studies (Approval # 2011–026), which were also supervised by a veterinarian.

### Animals

Male wild-type C57Bl/6, *Nos2*^*−/−*^, *p47*^*phox−/−*^, and *gp91*^*phox−/−*^mice (8–10 weeks, 25–30 g; Charles River, St. Constant, Quebec) were randomized to volume-resuscitated CLP-sepsis under inhaled isoflurane anesthesia vs. sham surgery, as we previously described [17, 19, 37]. At 30 mins, 1, 2, and 4 h after surgery, mice were prepared for pulmonary IVVM or sacrificed using a lethal dose of s/c pentobarbital and exsanguination for lung formalin fixation, collection, and histologic slide preparation [37].

### Pulmonary microvascular albumin-permeability in vivo

Pulmonary microvascular albumin-permeability was assessed by the EB dye technique, as we previously described [18]. In brief, sham and CLP mice received tail vein injections of EB (50 μg/g) 30 mins prior to sacrifice at relevant time points. Following sacrifice, PBS-perfused lungs were harvested, formamide-extracted, EB content measured spectrophotometrically, and EB-albumin permeability calculated (μg EB/g lung/min).

### Pulmonary IVVM

Pulmonary IVVM was performed as we previously described [17]. In brief, anesthetized, tracheotomized, mechanically ventilated mice had a 10 mm diameter transparent window implanted in the right thoracic wall, permitting inverted microscopy and visualization of the sub-pleural pulmonary microcirculation (32 × 10.4 objective, DIAPHOT-300 epi-fluorescence microscope, Nikon, Melville NY) and digital recording (Pixelfly QE High Performance Monochrome Digital Camera System with ICX-285 CCD, Opticon, Kitchener, ON).

### Quantification of PMVEC Death/Apoptosis

PMVEC death was quantified using both IVVM and lung tissue histology, as we previously described [37]. In brief, propidium iodide (PI; 0.5 μg/g bodyweight; Sigma) was intravenously injected immediately prior to IVVM fluorescence microscopy imaging to label non-viable PMVEC (excitation/emission: 530/590 nm). IVVM results were confirmed using fluorescence microscopy on lung histological sections. All cell nuclei were stained (Hoechst 33342; excitation/emission: 346/460 nm), non-viable PI+ cells were quantified, and PMVEC were identified by labeling with rat anti-mouse CD31 (BD Pharmingen). In each of 3 lung sections per mouse, 3 random digital images were captured, and PI+/CD31+ PMVEC/high power field (hpf) were quantified.

To identify features of apoptosis in PMVEC, three different molecular markers were assessed using fluorescence microscopy on lung tissue sections. (1) Loss of cell membrane polarization (as indicated by presence of cell surface phosphatidylserine) was assessed by injecting mice with Annexin V (conjugated to Alexa Fluor 594; excitation/emission: 590/617 nm, Invitrogen, Burlington, Ontario) 30 mins prior to sacrifice and lung tissue harvest for subsequent imaging. (2) To detect caspase activation, mice were treated 30 mins prior to sacrifice with intravenous FAM-FLIVO (excitation/emission: 490/525 nm; Immunohistochemistry Technologies, Bloomington, Minnesota), which binds activated caspases. (3) Late-stage apoptotic DNA fragmentation was examined by terminal deoxynucleotidyl transferase dUTP nick end labeling (TUNEL; excitation/emission: 494/521 nm; *In Situ* Cell Death Detection, Roche, Laval, Quebec).

Separate cohorts of wild-type C57Bl/6 mice were treated intravenously with the broad-spectrum potent caspase-inhibitor quinoline-Val-Asp(Ome)-CH2-O-phenoxy (Q-VD, 1.0 μg/g bodyweight, APExBIO, Boston, Massachusetts) [44] or a highly specific pharmacologic inhibitor of NADPH oxidase (apocynin, 20 μg/g bodyweight, Abcam, Toronto, Ontario) [45] 30 mins prior to CLP-septic vs. sham procedures.

### Quantification of Pulmonary Microvascular Polymorphonuclear (PMN) Leukocyte Sequestration

As we previously described, rhodamine-6-G-positive PMN were quantified (rhodamine-6-G excitation/emission: 530/580 nm) [17, 18, 37]. Digital images were obtained and rhodamine-6-G-positive cells were quantified.

### Quantification of CLP-induced peritonitis

As in human sepsis and related ALI/ARDS, this murine CLP model of septic ALI depends critically on the intensity of the initial infectious focus, the CLP-induced peritonitis. As experimental interventions, such as use of genetically deficient mouse strains or pharmacologic antagonists, could conceivably modify the initial peritoneal infection or inflammatory response, the degree of peritonitis following CLP vs. sham surgery was assessed using 3 parameters, peritoneal wash bacterial concentration, total leukocyte counts, and protein levels, as previously reported [19, 46].

### Statistical analysis

Data are mean ± SEM. Between-group differences were assessed by *t*-test, one-way ANOVA with Tukey post-hoc test, or two-way ANOVA with Bonferroni post-hoc test, where appropriate. *p* < 0.05 was accepted as significant. The correlation of time-dependent changes in pulmonary microvascular EB-albumin permeability with PMVEC death (PI+) or apoptosis (FLIVO+) was assessed by Pearson’s method.

## Results

### Time Course of Sepsis-induced PMVEC Barrier Dysfunction and Death

**Fig. 1:**
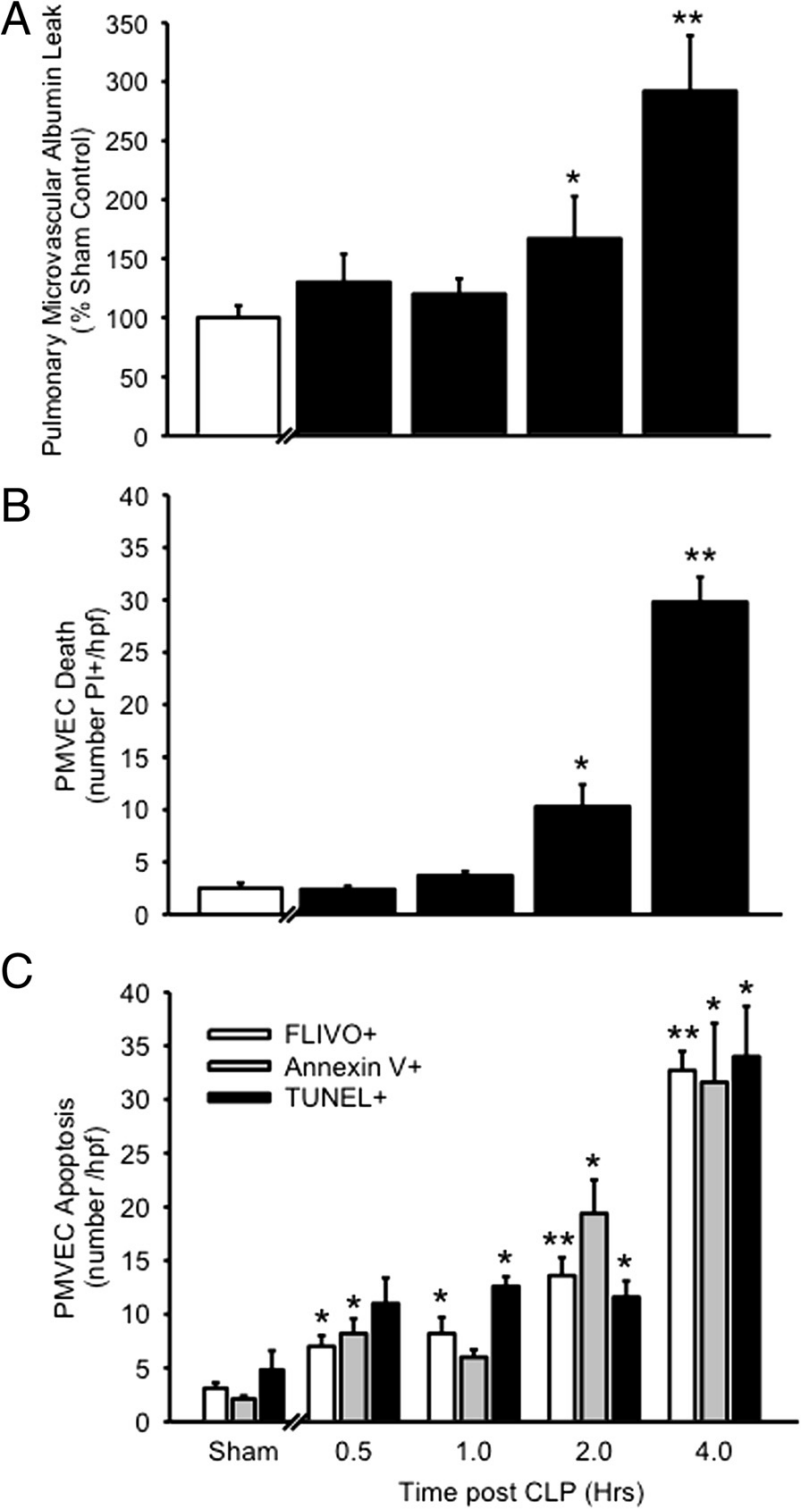
Time course of pulmonary microvascular endothelial cell (PMVEC) albumin-permeability barrier dysfunction, death and apoptosis following cecal ligation/perforation (CLP)-sepsis in mice *in vivo*. **a** The leak of Evans blue (EB) dye-labeled albumin into lung tissue increased 2 and 4 h after CLP-induced sepsis, compared to sham control mice. *n* = 14 (Sham) and *n* = 3–10 /time point (CLP). **b** PMVEC death, as assessed through nuclear propidium iodide (PI+) staining visualized by fluorescence intravital videomicroscopy, was observed 2 and 4 h after CLP-induced sepsis, compared to sham control mice. *n* = 6 (Sham; combined 0.5 and 4 h time points) and *n* = 3–9 /time point (CLP). **c** PMVEC apoptosis was quantified by 3 complementary molecular markers: phosphatidyl serine expression (Annexin V+ staining), caspase activation (FLIVO+ staining), and DNA fragmentation (TUNEL+ labeling). Compared to low-level PMVEC apoptosis in sham control mice, enhanced septic PMVEC apoptosis was observed 0.5 – 1 h after CLP-induced sepsis and increased in time-dependent manner until 4 h. *n* = 6 (Sham; combined 0.5 and 4 h time points) and *n* = 3–9 /time point (CLP). *p < 0.05 and **p < 0.01 for CLP vs. sham group

As previously reported [17, 19], the murine CLP-model of sepsis is characterized by significant clinical illness, as septic mice exhibit huddling, piloerection, tachypnea, and reduced exploratory behaviour. We first defined the time course of early changes in pulmonary microvascular permeability *in vivo* over the first 30 mins – 4 h after CLP-induced sepsis vs. sham surgery (Fig. 1a). In sham mice, basal pulmonary microvascular endothelial permeability, as reflected by lung tissue content of EB-dye-labeled albumin, was 1.1 ± 0.2 μg EB/g lung/min, which was not significantly different from 30 mins – 4 h after sham procedures (data not shown). Immediately after CLP-sepsis surgery (30 mins), pulmonary microvascular endothelial EB-albumin permeability was slightly, but not significantly greater than in sham mice, at 1.4 ± 0.3 μg EB/g lung/min. By 2 h post-CLP, septic mice had evidence of significant pulmonary microvascular endothelial barrier dysfunction compared to sham-treated mice, and this difference was more marked at 2.7 ± 0.4 μg EB/g lung/min by 4 h after CLP-sepsis (Fig. 1a).

We next assessed the time course of septic PMVEC death *in vivo* with pulmonary IVVM and *ex vivo* in lung tissue sections over the first 30 mins – 4 h after CLP-induced sepsis vs. sham surgery (Fig. 1b). In sham mice, there was a low basal rate of PMVEC death (PI+ staining; 2.5 ± 0.5 cells/hpf, and 99 ± 1 % of these non-viable cells were PMVEC, identified through PI+/CD31+ double-staining overlap. Overall, 8 ± 1 % of CD31+ PMVEC were also PI+ in sham mice, and this low basal level of PMVEC death was similar between 30 mins and 4 h after sham surgery (data not shown). Following CLP-induced sepsis, PMVEC death (number PI+/hpf) was significantly increased at 2 and 4 h after CLP-sepsis compared to sham. As in sham mice, these PI+/dead cells were almost uniformly PMVEC, based on PI+/CD31+ double-staining overlap in 95 ± 3 %. In contrast to sham mice, PI+/dead PMVEC represented a sizable proportion, 71 ± 5 %, of all PMVEC/hpf at 4 h after CLP-sepsis.

**Fig. 2:**
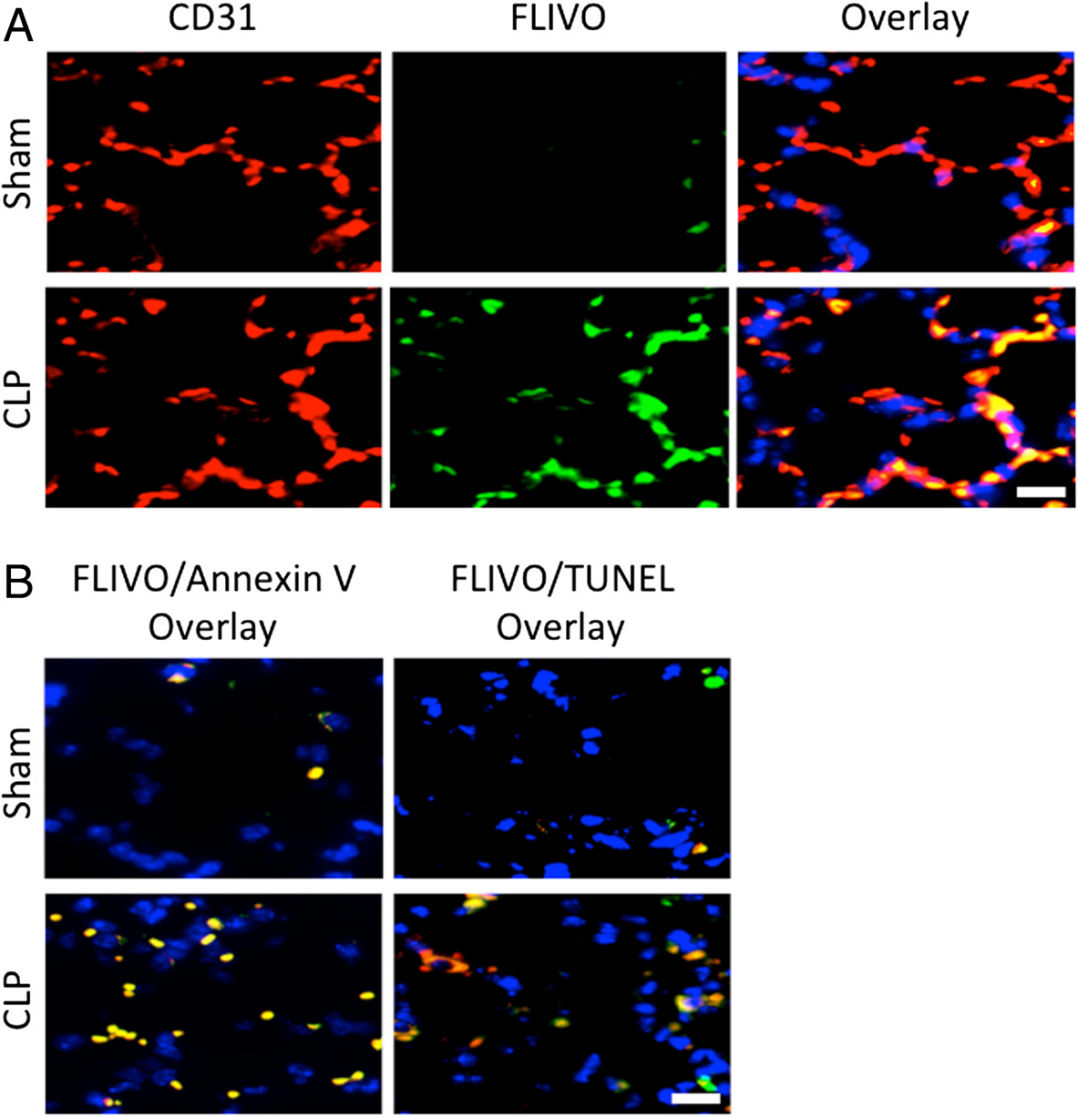
Evidence of PMVEC apoptosis 4 h following CLP-sepsis in mice *in vivo*. **a** PMVEC in *ex vivo* lung tissue sections were labeled with a specific marker (CD31; left column of panels; red). Activated caspases were identified by labeling with FLIVO (middle column of panels; green). Compared to sham mice in which only a few PMVEC stained positive for activated caspases, CLP-sepsis markedly increased the number PMVEC with activated caspases (overlay; right column of panels). **b** Compared to sham mice, CLP-sepsis was associated with increased PMVEC apoptosis as identified using three complementary markers: FLIVO (activated caspases), Annexin V (surface phosphatidyl serine presence), and TUNEL (DNA fragmentation). At 4 h following CLP-sepsis, FLIVO+ PMVEC (green) were almost uniformly also Annexin V+ (left column of panels), and were largely but not always TUNEL+ (right column of panels). Scale bar = 20 μm

Septic PMVEC death (PI+ staining) post-CLP appeared to be almost completely due to PMVEC apoptosis, based on fluorescent microscopy of lung tissue sections. The earliest measurable apoptotic signals were increases in both PMVEC FLIVO staining (caspase activation) and Annexin V staining at 30 mins after CLP-sepsis (Fig. 1c). PMVEC apoptosis appears to have increased progressively until 4 h after CLP-sepsis, at which time point there was strong evidence of septic PMVEC apoptosis as quantified by all 3 molecular markers of apoptosis: enhanced Annexin V binding, FLIVO staining, and TUNEL presence. There was a high degree of overlap between the 2 earliest markers of apoptosis (Annexin V binding and FLIVO staining) at 4 h after CLP-sepsis (Fig. 2): 100 ± 0 % of Annexin V+ PMVEC were also FLIVO+, and 93 ± 3 % of FLIVO+ PMVEC were Annexin V+. In contrast, only 74 ± 5 % of TUNEL+ PMVEC were also FLIVO+, and conversely, 65 ± 1 % of FLIVO+ PMVEC were also TUNEL+.

### Role of PMVEC Death/Apoptosis in Sepsis-Induced PMVEC Barrier Dysfunction

**Fig. 3:**
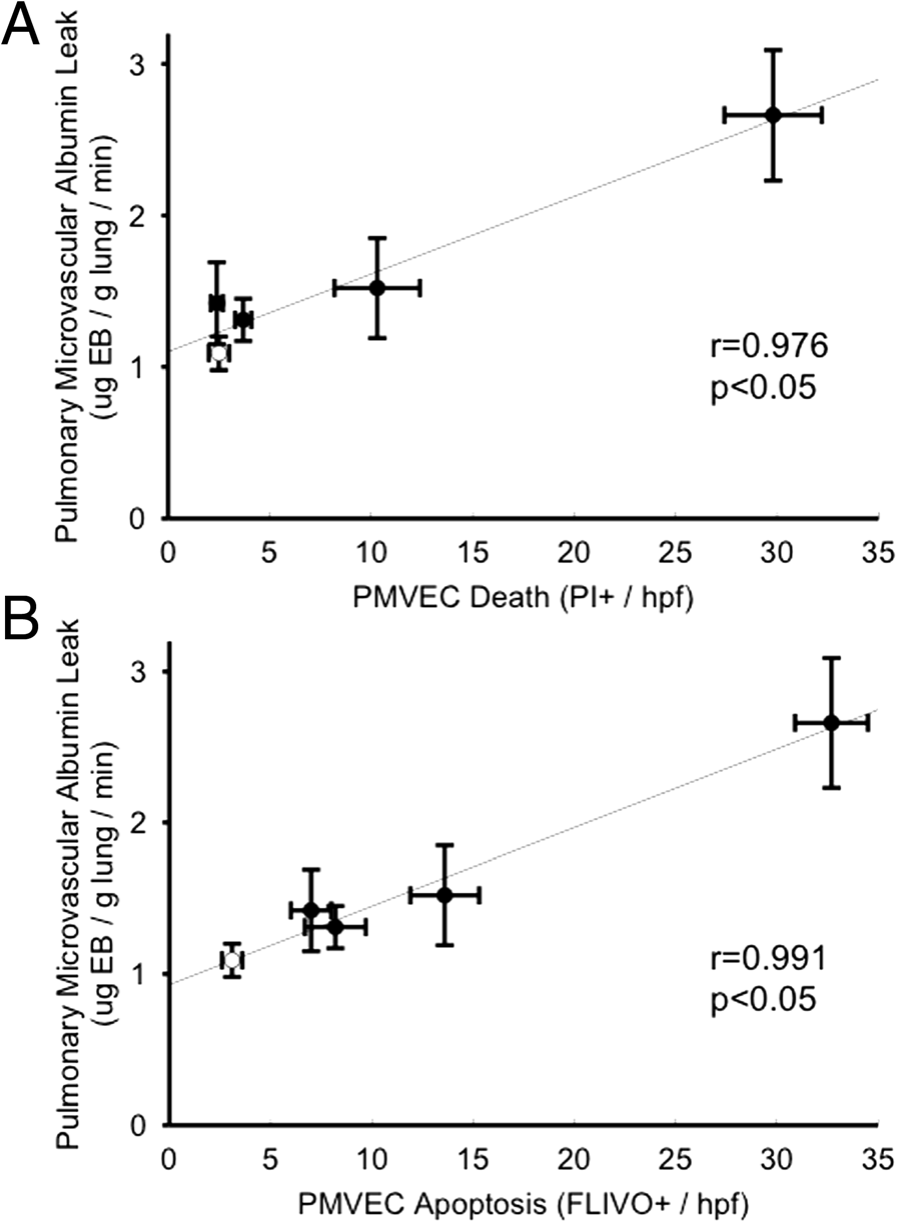
Correlation of time-dependent changes in septic PMVEC albumin-permeability barrier dysfunction with (**a**) PMVEC death (PI+ staining) and (**b**) PMVEC apoptosis (FLIVO+ staining) following CLP-sepsis in mice *in vivo*. Open symbols (sham, *n* = 6), filled symbols (CLP, *n* = 3–9 /time point)

Following CLP-sepsis, the time-dependent disruption of pulmonary microvascular/PMVEC barrier function, as reflected by pulmonary microvascular EB-albumin permeability *in vivo*, was strongly correlated (*r* = 0.976, *p* < 0.05; Fig. 3a) with IVVM PMVEC death (PI+). Moreover, pulmonary microvascular EB-albumin permeability was also highly correlated specifically with histologic markers of PMVEC apoptosis, including caspase activation (FLIVO+ staining; *r* = 0.991, *p* < 0.05; Fig. 3b), Annexin V+ labeling (*r* = 0.938, *p* < 0.05), and TUNEL+ labeling (*r* = 0.987, *p* < 0.05).

**Fig. 4:**
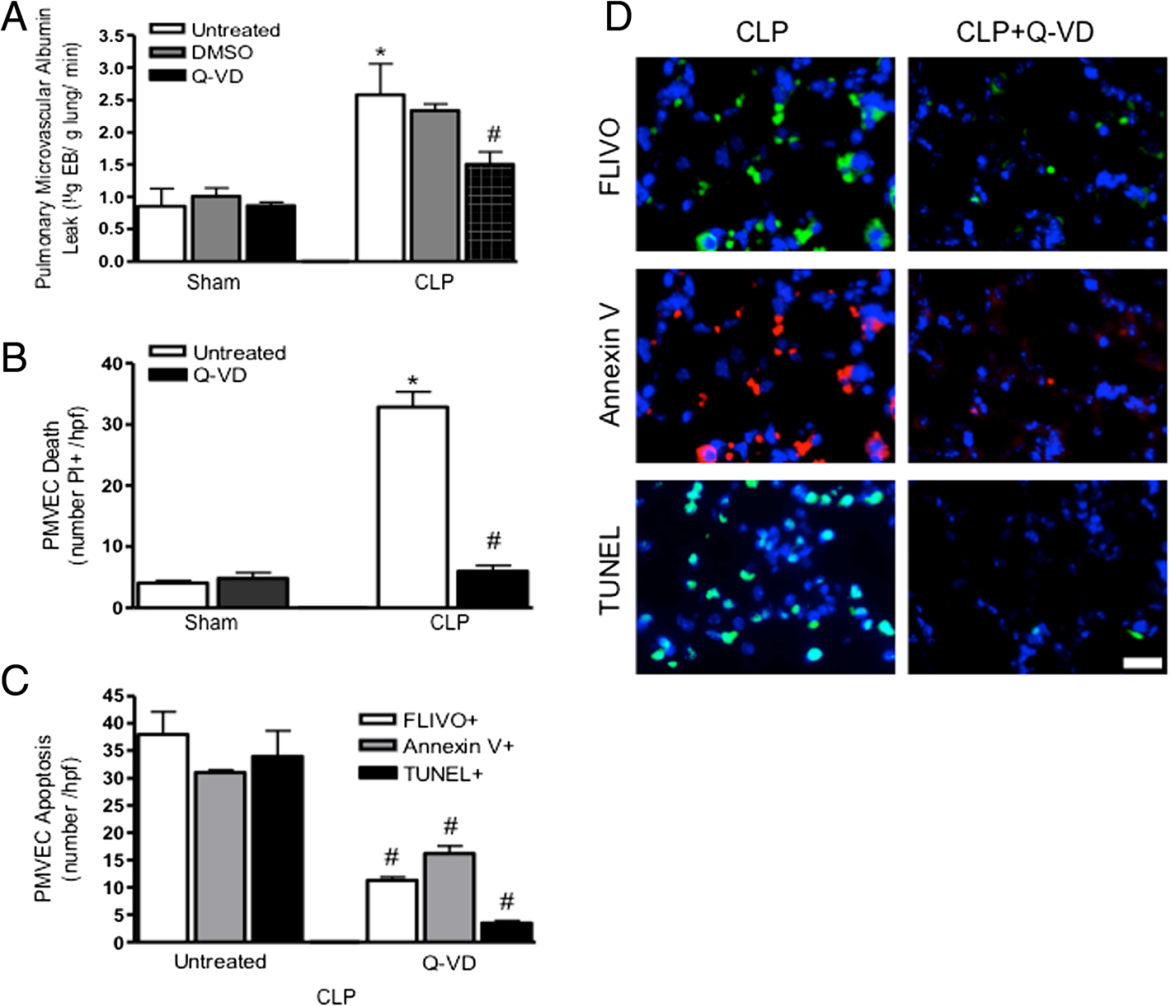
Role of PMVEC apoptosis in septic pulmonary microvascular permeability barrier dysfunction. **a** Treatment of wild-type mice with the multi-caspase inhibitor Q-VD inhibited septic pulmonary microvascular EB-albumin leak at 4 h following CLP-sepsis. Q-VD treatment also markedly attenuated septic PMVEC death (PI+; panel **b**) and PMVEC apoptosis (FLIVO+, Annexin V+, TUNEL+; panels **c** and **d**) at 4 h following CLP-sepsis. *n* = 3-4 (Sham) and *n* = 4-6 (CLP). * *p* < 0.05, CLP vs. respective sham group; #, *p* < 0.05 Q-VD-treated vs. untreated CLP. Scale bar = 20 μm

The specific role of septic PMVEC apoptosis in septic pulmonary microvascular/PMVEC barrier dysfunction *in vivo* was pursued by inhibiting apoptosis using the broadly-selective, irreversible pharmacologic antagonist of activated caspases, Q-VD. Treatment of sham mice with either Q-VD or the DMSO solvent control had no effects on the normal pulmonary microvascular/PMVEC EB-albumin permeability *in vivo* or the low, basal levels of PMVEC death (PI+) or apoptosis (FLIVO+, Annexin V+, TUNEL+; Fig. 4). In contrast, Q-VD treatment prior to CLP-sepsis surgery significantly attenuated septic pulmonary microvascular/PMVEC permeability barrier dysfunction (Fig. 4a) as well as septic increases in both PMVEC death (decreased PI+ staining; Fig. 4b) and specifically apoptosis as reflected by decreased presence of FLIVO, Annexin V, and TUNEL labeling (Fig. 4c and d).

### Role of iNOS and NADPH oxidase in Sepsis-induced PMVEC apoptosis

**Fig. 5:**
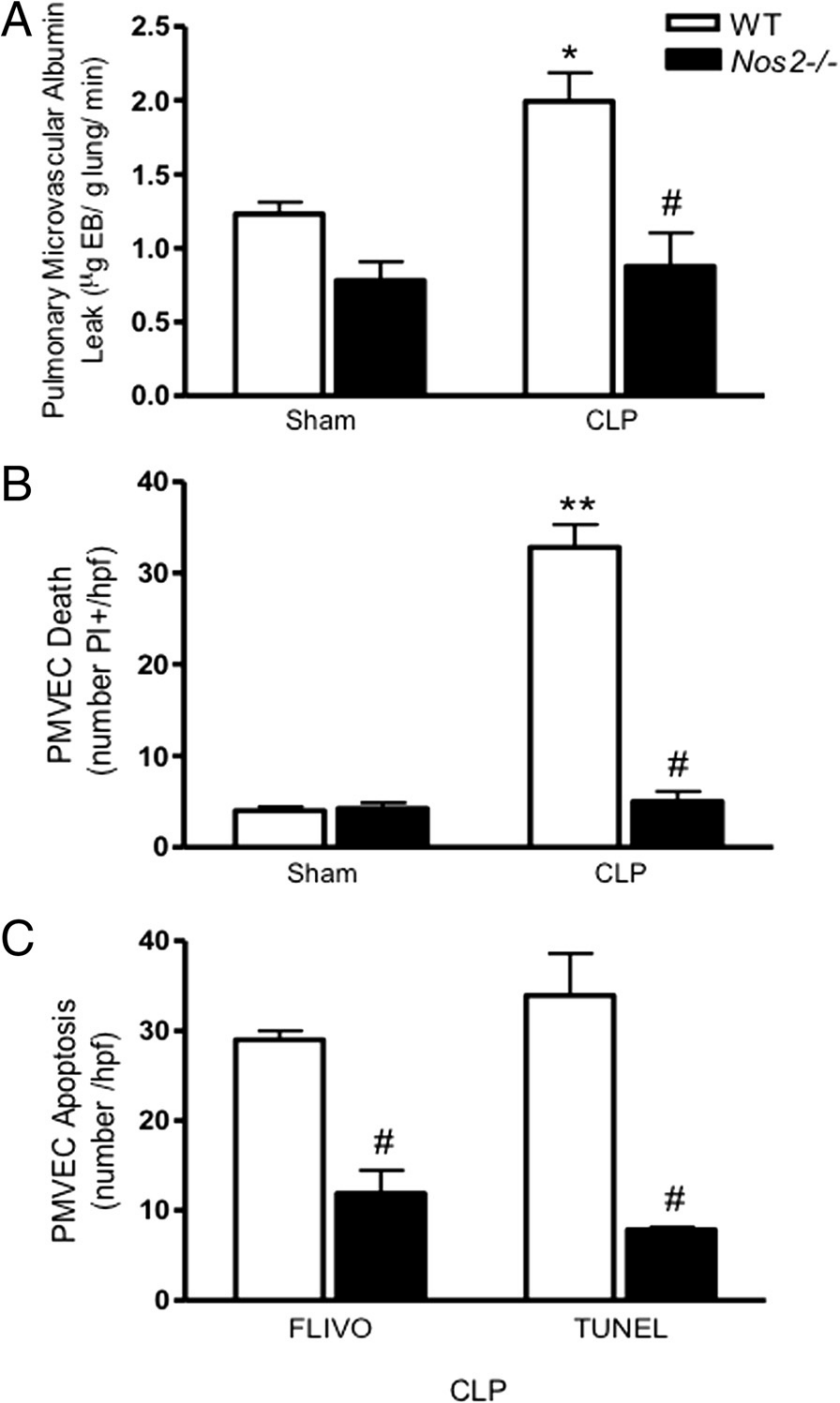
Role of iNOS in septic PMVEC permeability barrier dysfunction, death and apoptosis 4 h following CLP-sepsis in mice *in vivo*. As compared to septic wild-type (WT) mice, iNOS deficiency (*Nos2* ^*−/−*^) was associated with complete attenuation of septic increases in (**a**) pulmonary microvascular EB-albumin leak, (**b**) PMVEC death, and (**c**) PMVEC apoptosis (FLIVO+, TUNEL+). *n* = 3–4 (Sham) and *n* = 3–6 (CLP). * *p* < 0.05 and ** *p*<0.01, CLP vs. respective sham group; # *p* < 0.05, *Nos2* ^*−/−*^ vs. wild-type CLP

We have previously reported that iNOS plays an important role in multiple features of septic ALI in the murine CLP-sepsis model, including pulmonary microvascular endothelial barrier dysfunction and the resulting albumin hyper-permeability, pulmonary oxidant stress, pulmonary microvascular PMN sequestration and trans-PMVEC PMN migration [17, 18, 35, 47]. Thus, we assessed the contribution of iNOS to septic PMVEC apoptosis in CLP-sepsis vs. sham mice. We first confirmed that septic pulmonary microvascular/PMVEC barrier dysfunction was prevented in mice lacking iNOS (*Nos2*^*−/−*^) undergoing CLP surgery (Fig. 5a). Similarly, septic PMVEC death (PI+) in wild-type mice was also abrogated in septic *Nos2*^*−/−*^ mice (Fig. 5b). Furthermore, reduced PMVEC FLIVO+ and TUNEL+ staining (Fig. 5c) in septic *Nos2*^*−/−*^ mice confirm that the reduction in PMVEC death was specifically due to a decrease in PMVEC apoptosis in septic *Nos2*^*−/−*^ mice vs. wild-type mice.

**Fig. 6:**
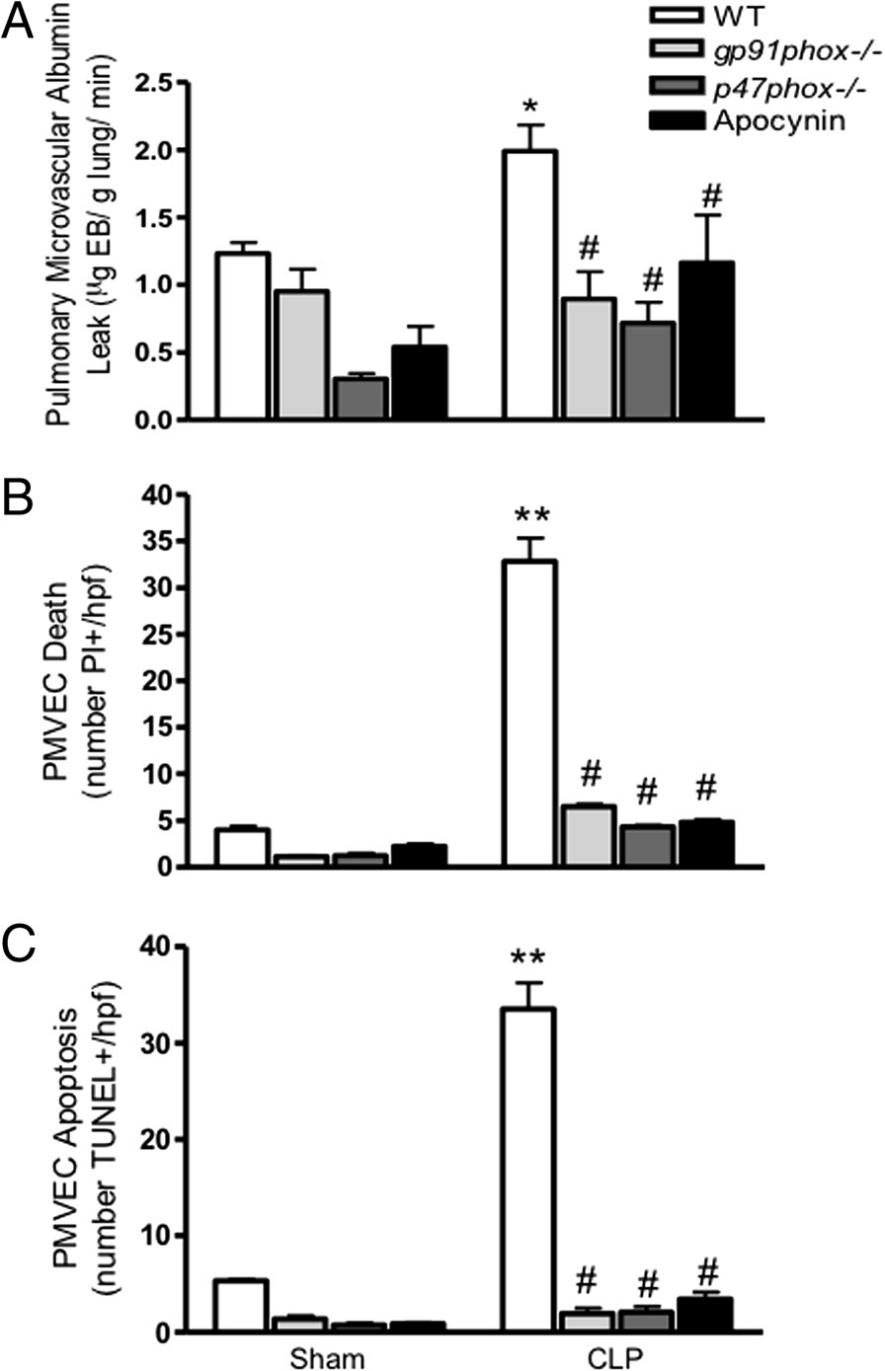
Role of NADPH oxidase in septic PMVEC permeability barrier dysfunction, death and apoptosis 4 h following CLP-sepsis in mice *in vivo*. As compared to septic WT mice, NADPH oxidase deficiency (both *gp91* ^*phox−/−*^ and *p47* ^*phox−/−*^) or inhibition (apocynin) was associated with complete attenuation of septic increases in (**a**) pulmonary microvascular EB-albumin leak, (**b**) PMVEC death, and (**c**) PMVEC apoptosis (TUNEL+). *n* = 3–4 (Sham) and *n* = 3–6 (CLP). * *p* < 0.05 and ** *p*<0.01, CLP vs. respective sham group; #, *p* < 0.05 vs. wild-type CLP

Similarly, the role of NADPH oxidase in septic pulmonary microvascular/PMVEC barrier dysfunction and PMVEC death/apoptosis was defined by comparing septic responses in wild-type mice vs. mice deficient in functional NADPH oxidase (*gp91*^*phox−/−*^ and *p47*^*phox−/−*^). Compared to CLP-septic wild-type mice, septic pulmonary microvascular/PMVEC EB-albumin permeability barrier dysfunction *in vivo* was attenuated in septic *gp91*^*phox−/−*^ and *p47*^*phox−/−*^ mice (Fig. 6a). In parallel, septic PMVEC death (PI+ staining; Fig. 6b) and apoptosis (TUNEL+ staining; Fig. 6c), were also inhibited in septic *gp91*^*phox−/−*^ and *p47*^*phox−/−*^ vs. wild-type mice. Moreover, the role of NADPH oxidase was confirmed by quantifying the effects of NADPH oxidase pharmacologic inhibition using apocynin. Pre-treatment of wild-type mice with apocynin prior to CLP-sepsis surgery similarly abrogated the septic increases in pulmonary microvascular/PMVEC EB-albumin permeability, PMVEC death (PI+) and apoptosis (TUNEL+) (Fig. 6a, b, c), similar to the observations in septic NADPH oxidase-deficient mouse strains. Apocynin inhibition of CLP-septic PMVEC apoptosis was further confirmed by demonstrating complete attenuation of septic PMVEC FLIVO+ staining (3 ± 1 cells /hpf, *p* < 0.05 vs. septic wild-type) and Annexin V+ staining (3 ± 1 cells /hpf, *p* < 0.05 vs. septic wild-type), with 99 ± 1 % overlap between the 2 apoptosis markers.

### Effects of Sepsis and PMVEC Apoptosis on Pulmonary Microvascular PMN Sequestration

**Fig. 7:**
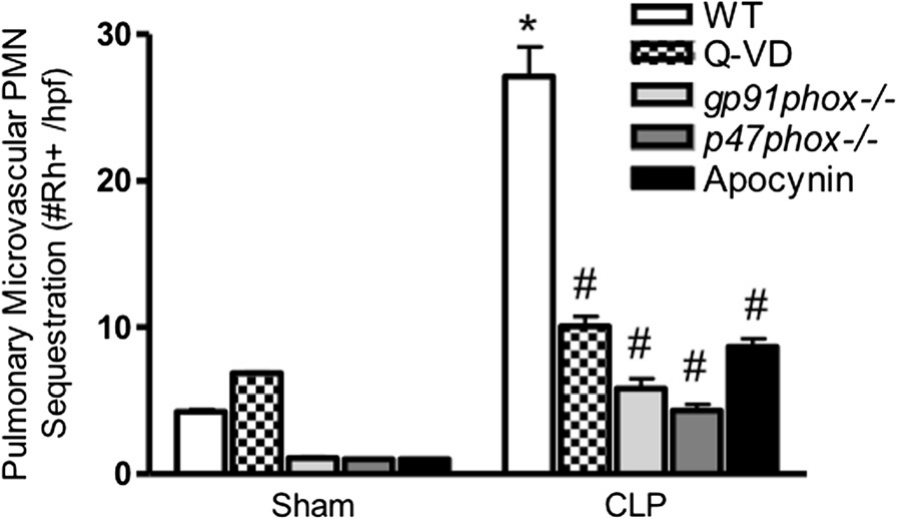
Effects of CLP-sepsis, Q-VD treatment (caspase inhibition), and NADPH oxidase deficiency or inhibition on pulmonary microvascular polymorphonuclear leukocyte (PMN) sequestration in murine sepsis *in vivo*. In septic WT mice, pulmonary microvascular/PMVEC dysfunction is associated with dramatic increases in pulmonary microvascular PMN sequestration, as assessed by rhodamine-6-G (Rh+) labeling and visualization by fluorescence IVVM. This septic pulmonary microvascular / PMVEC dysfunction and resulting increased PMN sequestration were markedly attenuated following caspase inhibition (Q-VD treatment in CLP-WT mice), in septic *gp91* ^*phox−/−*^ and *p47* ^*phox−/−*^ mice, and following NADPH oxidase inhibition (apocynin treatment in CLP-WT mice). *n* = 2-3 (Sham) and *n* = 3-4 (CLP). * *p* < 0.05, CLP vs. respective sham group; #, *p* < 0.05 vs. WT CLP

We have previously demonstrated that pulmonary microvascular dysfunction in the murine CLP-sepsis model is also characterized by significantly enhanced pulmonary microvascular PMN sequestration [17, 37]. Thus, we further examined the relationship between this CLP-septic pulmonary microvascular PMN sequestration and septic PMVEC death/apoptosis. We confirmed our previous reports of a marked increase in the number of rhodamine-6-G-labeled PMN sequestered in the pulmonary microvasculature *in vivo* (by IVVM; Fig. 7) in CLP-septic wild-type vs. sham mice, and confirmed this *ex vivo* by fluorescence microscopy in lung histologic sections (data not shown). This feature of septic pulmonary microvascular dysfunction, specifically enhanced PMN sequestration, was significantly attenuated following: (a) inhibition of caspases with Q-VD pre-treatment of wild-type mice prior to induction of CLP-sepsis, (b) CLP-sepsis in *gp91*^*phox−/−*^ and *p47*^*phox−/−*^ mice, and (c) inhibition of NADPH oxidase with apocynin pre-treatment of wild-type mice prior to CLP-sepsis. Similarly, we have previously reported that this CLP-septic pulmonary microvascular PMN sequestration was inhibited in septic *Nos2*^−/−^ vs. wild-type mice [17, 37].

### CLP-Induced Peritonitis and Effects of Experimental Interventions

CLP surgery was associated with purulent peritonitis on gross inspection, which was confirmed by significant bacterial presence and quantitative increases in peritoneal protein levels and total cell counts in all CLP-septic groups vs. respective sham groups (Table 1). CLP-sepsis resulted in slightly lower peritoneal total cell counts in Q-VD treated vs. control DMSO-treated CLP-septic mice. Otherwise, the intensity of CLP-induced peritoneal infection and inflammation was not significantly different between control DMSO-treated CLP-septic mice, pharmacologically-treated CLP-septic groups (Q-VD, apocynin), or CLP-septic *gp91*^*phox−/−*^ and *p47*^*phox−/−*^ groups. We have previously reported similar data on the lack of any difference in CLP-induced peritonitis between septic wild-type and *Nos2*^−/−^ mice [19].

**Table 1:** Effect of CLP-sepsis and various experimental interventions on severity of peritonitis

Animal/Treatment Group		Peritoneal Lavage Parameter					
		Sham			CLP		
		Protein	Bacterial load	Total Cells	Protein	Bacterial load	Total Cells
		*(mg/mL)*	*(10* ^*4*^ *CFU/mL)*	*(10* ^*7*^ */mL)*	*(mg/mL)*	*(10* ^*4*^ *CFU/mL)*	*(10* ^*7*^ */mL)*
Wild-Type							
	DMSO	0.6 ± 0.1	0.0 ± 0.0	0.07 ± 0.01	2.2 ± 0.2	5.4 ± 0.7	3.6 ± 0.2
	Q-VD	0.7 ± 0.1	0.0 ± 0.0	0.07 ± 0.01	2.1 ± 0.1	5.4 ± 1.1	2.5 ± 0.1 *
NADPH oxidase deficient							
	p47phox−/−	1.0 ± 0.3	0.3 ± 0.3	0.12 ± 0.04	2.4 ± 0.5	5.9 ± 1.2	3.3 ± 0.3
	gp91−/−	1.1 ± 0.4	0.2 ± 0.3	0.15 ± 0.05	2.6 ± 0.7	6.4 ± 1.5	2.9 ± 0.4

*, *p* < 0.05 vs. DMSO control CLP group

CFUs indicate colony-forming units

## Discussion

In the murine CLP-septic ALI model, pulmonary microvascular/PMVEC barrier dysfunction *in vivo* and resulting albumin hyper-permeability developed rapidly over 2–4 h after CLP-sepsis, with a similar time course as PMVEC death, as reflected by PI+ staining, and with specific features consistent with PMVEC apoptosis, including surface phosphatidyl serine presence (Annexin V staining), caspase activation (FLIVO staining), and DNA fragmentation (TUNEL labeling). Moreover, septic pulmonary microvascular/PMVEC barrier dysfunction correlated strongly with measures of PMVEC death/apoptosis. Pharmacologic caspase inhibition with Q-VD *in vivo* prior to CLP significantly prevented septic PMVEC death/apoptosis and attenuated septic pulmonary microvascular/PMVEC dysfunction, as reflected by both reduced albumin-permeability barrier dysfunction and pulmonary microvascular PMN sequestration. Septic PMVEC apoptosis and microvascular/PMVEC dysfunction *in vivo* were both similarly abolished following CLP-sepsis in mice lacking iNOS (*Nos2*^*−/−*^) or NADPH oxidase activity (*p47*^*phox−/−*^ and *gp91*^*phox−/−*^), as well as in wild-type mice pre-treated with the NADPH oxidase pharmacologic inhibitor, apocynin. Thus, septic pulmonary microvascular/PMVEC dysfunction including high-albumin pulmonary edema and enhanced PMN sequestration, key pathophysiologic features of septic ALI, appear to be the result of PMVEC death specifically due to caspase-dependent apoptosis, which appears to be mediated through the activity of both iNOS and NADPH oxidase.

The high morbidity and mortality of sepsis are the result of multiple organ dysfunction/failure, including ALI/ARDS, which is due in large part to septic injury and dysfunction of the microvasculature and especially MVEC [2, 4, 5, 48]. Septic microvascular/MVEC injury and dysfunction are characterized pathophysiologically by perturbed microvascular distribution of blood flow in many organs, enhanced microvascular thrombosis, enhanced PMN-MVEC interaction, and disruption of the normal microvascular/MVEC permeability barrier, resulting in high-permeability albumin-rich tissue and organ edema [13, 14, 17–21, 30, 48–53]. The severe hypoxaemic respiratory failure of ALI/ARDS is pathophysiologically due to pulmonary microvascular PMN sequestration and influx into pulmonary interstitial and alveolar compartments, mediated through enhanced PMN-PMVEC adhesion, and the associated injury and dysfunction of the pulmonary microvascular alveolo-capillary permeability barrier and resulting extra-vascular leak of protein-rich edema [17–21, 51].

In ALI/ARDS, pathologic disturbances have been described in the structure and function of both key cells in the normal alveolo-capillary permeability barrier, namely PMVEC and alveolar epithelial cells (AEC) [54, 55]. For example, AEC dysfunction in ALI/ARDS is characterized by impairment of their normal barrier and alveolar water clearance functions, which is associated with worse outcome in ARDS patients [56]. Moreover, AEC also contribute to inflammation in ALI/ARDS, as they are an important source of cytokines and chemokines [54], and also foster intra-alveolar coagulation [57, 58]. Human AEC may also have a critical role in protecting adjacent human PMVEC against septic injury through a paracrine mechanism, as we recently described in human PMVEC-AEC co-cultures *in vitro* [59].

Most importantly in sepsis, injury and dysfunction of the pulmonary microvasculature and specifically PMVEC are central to the pathophysiology of septic ALI. Our previous work has established in murine CLP-sepsis that pulmonary microvascular/PMVEC permeability barrier dysfunction requires the presence of PMN and alveolar macrophages, PMN-PMVEC CD18-dependent adhesion, as well as functional iNOS and NADPH oxidase [17–19, 34–36]. Moreover, this *in vivo* murine CLP-septic ALI model is characterized by pulmonary oxidant and specifically nitrosative stress, consistent with NO-superoxide reaction and peroxynitrite generation [35]. Consistent with the *in vivo* findings, cultured murine and human PMVEC under septic conditions exhibit permeability barrier dysfunction, which is similarly enhanced by the presence of PMN and macrophages in co-culture, requires PMN-PMVEC CD18-dependent adhesion and specifically the presence of functional iNOS in PMN and macrophages [60–64]. Most recently, we pursued the potential critical role of putative PMVEC death in the CLP-septic murine model *in vivo* [37].

The death of cells in pathologic conditions is an increasingly important and complex research topic, as there are now as many as 10 different mechanisms, e.g. apoptosis, necrosis, necroptosis, pyroptosis, and autophagy [65, 66]. The specific mechanisms through which cells ultimately die depend upon many factors, including the nature of the initial injurious stimulus, the specific cell type, and the time point assessed, and are best identified by specific molecular markers (e.g. caspase 3/7 activation and DNA fragmentation [TUNEL+] with apoptosis, caspase 1 activation in pyroptosis, DNA fragmentation in the absence of caspase activation in necrosis) [65–67]. The death of EC can be through many of these mechanisms, although evidence for many mechanisms is largely derived from study *in vitro*. For instance, bacterial infection is associated with increased autophagy, sepsis can be associated with evidence of both apoptosis and necrosis, and inflammation following blood transfusion or lung infection has been found to cause necroptosis as well as pyroptosis [37, 68–71].

Although it is often stated that there is enhanced EC death in sepsis, this remains controversial, as evidence is relatively sparse, especially direct *in vivo* data in clinically relevant models of sepsis (e.g. CLP). Few studies have reported quantification of apoptotic EC specifically under septic conditions [38, 41, 43, 72], possibly due to the shedding of such apoptotic EC from the microvasculature [39]. Moreover, various septic models have been used, e.g. intravenous or intra-peritoneal LPS administration, some of which may not capture the complex pathophysiology of bacterial sepsis and septic ALI. In addition, identification of apoptotic cells has often been with a single marker of apoptosis (e.g. TUNEL staining), and confirmation of EC-specific apoptosis using double-staining with EC-specific markers has rarely been reported. Specifically in humans, there is limited direct evidence for septic EC death/apoptosis, which is largely supported by increased levels of circulating ECs and plasma caspase 3 [73, 74].

In the murine CLP-sepsis model, we recently reported significant septic PMVEC death (PI+ PMVEC confirmed through double-labeling with CD31, CD34, and *G. simplicifolia* lectin) as observed by IVVM and confirmed by pulmonary histology [37]. Moreover, septic PMVEC death appeared to be largely due to PMVEC apoptosis, as reflected by high degrees of overlap on double-staining with increased Annexin V binding, FLIVO (caspase activation) and TUNEL (DNA fragmentation) staining. However, the putative direct role of PMVEC death in septic ALI, specifically *in vivo* pulmonary microvascular/PMVEC dysfunction, had not yet been clearly defined.

Cell death can be both beneficial (promotes recovery and prevents injury propagation) and detrimental (promotes loss of tissue function and inhibits repair), dependent again on the nature of the injury and the cell type. Death of EC, especially MVEC, has been proposed as an important mechanism of septic microvascular/MVEC dysfunction. In the current study, three lines of evidence solidly support an essential role of PMVEC death/apoptosis in murine septic pulmonary microvascular/PMVEC dysfunction *in vivo*: (a) following CLP-sepsis, pulmonary microvascular/PMVEC barrier dysfunction and PMVEC death/apoptosis followed parallel time courses; (b) there were strong correlations between *in vivo* septic pulmonary microvascular/PMVEC barrier dysfunction and both PMVEC death (PI+) and markers of apoptosis (e.g. FLIVO+); and (c) irreversible pan-caspase pharmacologic inhibition with Q-VD markedly blunted septic PMVEC death/apoptosis and attenuated *in vivo* septic pulmonary microvascular/PMVEC dysfunction, including both albumin-permeability barrier dysfunction and pulmonary microvascular PMN sequestration.

Our previous work and the present study suggest caspase-dependent apoptosis as the most likely mechanism of PMVEC death in murine sepsis. The specific and rigorous characterization of apoptosis is important, as individual molecular markers commonly used to define apoptosis can be present during non-apoptotic cell death and even under inflammatory non-death conditions. In fact, it is widely accepted that apoptosis should be defined using multiple molecular markers [65, 66], and thus, in the current study, three distinct markers were used to define apoptosis: Annexin V (surface phosphatidylserine localization), FLIVO (caspase activation), and TUNEL labeling (DNA fragmentation). Furthermore, the effects of the potent caspase pharmacologic inhibitor Q-VD in markedly attenuating PMVEC apoptosis (by all three markers) confirms our putative role for PMVEC caspase-dependent apoptosis in PMVEC death and septic pulmonary microvascular/PMVEC dysfunction. Our findings are consistent with previous work on the role of caspase activation in EC apoptosis *in vivo* [42, 43, 75].

The present study also demonstrated that both iNOS and NADPH oxidase expression/activity were essential to murine septic PMVEC death/apoptosis, and the resulting *in vivo* pulmonary microvascular/PMVEC dysfunction. Septic upregulation of iNOS expression and activity have been recognized to contribute to pulmonary cell apoptosis [76]. For example, both *Nos2*^*−/−*^ mice and iNOS-inhibited wild-type mice had decreased numbers of apoptotic alveolar and bronchiolar epithelial cells following sepsis [76]. The vital chemical interplay between these 2 key oxidant mediator systems, iNOS-NO and NADPH oxidase-superoxide, has long been recognized in sepsis and ALI pathophysiology. NO and superoxide react extremely quickly to generate the highly potent oxidant peroxynitrite, which serves many physiologic and pathophysiologic functions [77, 78]. The central role of iNOS- and NADPH oxidase-signaling in septic PMVEC death/dysfunction in the present study is consistent with our previous data on septic murine pulmonary microvascular/PMVEC barrier dysfunction *in vivo* being associated with pulmonary oxidant and nitrosative stress [35]. Likewise, *in vitro* human PMVEC barrier dysfunction and PMN-PMVEC adhesion under septic conditions were also dependent on NO, superoxide, and peroxynitrite [63]. It is also clear that NADPH oxidase could contribute to apoptosis through multiple potential mechanisms, including through superoxide-dependent modulation of the expression of the cell cycle regulators, p21^cip1^ and p53, as well as through increased expression of CCAAT-enhancer-binding protein homologous protein (CHOP) [79, 80]. Additionally, we have shown that NADPH oxidase-dependent oxidant stress can mediate calpain-dependent caspase activity and apoptosis in septic murine PMVEC *in vitro* [40].

A central role for PMN in many of the pathophysiologic features of septic ALI in animal models, as well as in human ARDS has been clearly established [21, 81–83]. We and others have reported that septic pulmonary microvascular/PMVEC barrier dysfunction in mice *in vivo* and in murine and human PMVEC *in vitro* is clearly PMN-dependent [17, 18, 21, 35, 37, 60, 61, 84], and our recent study also strongly implicated pulmonary microvascular PMN leukocyte sequestration and CD18-dependent PMN-PMVEC adhesion in septic PMVEC death [37]. There are several contributory mechanisms of PMN-dependent pulmonary microvascular/PMVEC dysfunction including PMN-PMVEC physical interaction, release of PMN mediators (i.e. oxidants and proteases), and release of PMN extracellular traps [21, 85]. One of the key requirements for PMN-dependent pulmonary microvascular/PMVEC dysfunction appears to be the pulmonary microvascular PMN sequestration and PMN-PMVEC adhesion [17, 63, 84]. Intriguingly, in the present study, reduced PMVEC death/apoptosis in response to various interventions (e.g. Q-VD caspase inhibition, NADPH oxidase deficiency/inhibition) was associated with reduced pulmonary microvascular PMN sequestration, similar to our previous findings in septic *Nos2*^*−/−*^ mice [17, 37]. This suggests that septic PMVEC undergoing death/apoptosis may not be as immunologically “silent” or tolerogenic as initially thought [86], but may signal both adaptive immunity as well as paracrine inflammatory and pro-coagulant responses [87, 88].

We recognize the limitations of our study. Pulmonary microvascular barrier function is a complex system, and multiple mechanisms of septic ALI and specifically EC injury and dysfunction have been described in various animal models of sepsis/ALI *in vivo* and in isolated EC (including human EC models) under septic conditions *in vitro*. These include roles for altered cell-cell and cell-matrix (eg. glycocalyx) interactions, endogenous inflammatory cytokines (eg. TNFα) and vaso-active mediators (eg. vascular endothelial cell growth factor [VEGF], angiopoietins, endothelin), and cytoskeletal rearrangements [31–33]. We report that PMVEC death/apoptosis contribute importantly to septic pulmonary microvascular/PMVEC barrier dysfunction, but appreciate that our data do not exclude a role for other potential mechanisms that may also play a role, either directly or possibly indirectly through PMVEC death/apoptosis. For example, microvessel burn-induced decreased surface VE-cadherin localization and disrupted VEGF-signaling contribute to EC apoptosis, highlighting the potential cross-talk between various mechanisms of EC barrier dysfunction [89]. Similarly, it is clear that iNOS-dependent responses are complex and could contribute to pulmonary microvascular/PMVEC barrier dysfunction through other potential mechanisms, including modulation of inflammatory signaling (eg. NF-kB) and nitrosylation and/or nitration of multiple cell-surface and intracellular protein targets [34, 90, 91]. Finally, it should be noted that apocynin, a pharmacologic antagonist of NADPH oxidase, was used to confirm reduced PMVEC death/dysfunction in NADPH oxidase deficient mice, but apocynin also has anti-oxidant properties that may have contributed to its observed effects [92].

## Conclusions

We have shown for the first time in an *in vivo* murine septic ALI model that pulmonary microvascular/PMVEC dysfunction, resulting in microvascular PMN sequestration and albumin hyper-permeability *in vivo*, are due to septic PMVEC death and specifically caspase-dependent apoptosis. This septic PMVEC death/apoptosis and resulting pulmonary microvascular/PMVEC dysfunction were iNOS- and NADPH oxidase-dependent. Future studies will further define the contributory molecular pathways of septic PMVEC apoptosis and explore the potential clinical and therapeutic importance of septic PMVEC apoptosis in human sepsis/ARDS.
